# Liver lymphatic anatomy and role in systemic lymphatic disease

**DOI:** 10.1007/s00330-021-08098-z

**Published:** 2021-06-24

**Authors:** Christopher L. Smith, Mandi Liu, Madhumitha Saravanan, Aaron G. Dewitt, David M. Biko, Erin M. Pinto, Fernando A. Escobar, Ganesh Krishnamurthy, Jefferson N. Brownell, Petar Mamula, Andrew C. Glatz, Matthew J. Gillespie, Michael L. O’Byrne, Chitra Ravishankar, Jonathan J. Rome, Yoav Dori

**Affiliations:** 1 Jill and Mark Fishman Center for Lymphatic Disorders, The Children’s Hospital of Philadelphia, Philadelphia, PA 19104 USA; 2 Division of Cardiology, The Children’s Hospital of Philadelphia and Department of Pediatrics Perelman School of Medicine at The University of Pennsylvania, 3401 Civic Center Blvd, Philadelphia, PA 19104 USA; 3 Department of Radiology, The Children’s Hospital of Philadelphia and Perelman School of Medicine at The University of Pennsylvania, 3401 Civic Center Blvd, Philadelphia, PA 19104 USA; 4 Division of Gastroenterology, The Children’s Hospital of Philadelphia and Department of Pediatrics Perelman School of Medicine at The University of Pennsylvania, 3401 Civic Center Blvd., Philadelphia, PA 19104 USA

**Keywords:** Lymphatic diseases, Chylothorax, Ascites, Liver, Lymphography

## Abstract

**Objectives:**

To characterize hepatic to systemic lymphatic connections in patients with systemic lymphatic disease using intra-hepatic lymphangiography and to compare outcomes after lymphatic intervention.

**Methods:**

In this retrospective study, patients with intra-hepatic lymphangiography from May 2014 – April 2019 at our institution were included. Imaging review was performed and hepatic lymphatic connections and flow patterns were characterized. Clinical data were reviewed and comparisons between patients undergoing lymphatic intervention with or without abnormal hepatic lymphatics were performed.

**Results:**

During the study period, 105 patients underwent intra-hepatic lymphangiography. Primary clinical presentation included ascites (19/105), chylothorax (27/105), plastic bronchitis (PB) (17/105), and protein losing enteropathy (PLE) (42/105). Five categories of hepatic lymphatic connections and flow patterns were identified (%): normal (25%, 26/105), hepatoperitoneal (12%, 13/105), hepatopulmonary (10.5%, 11/105), hepatomesenteric (7.5%, 8/105), and hepatoduodenal (41%, 43/105) with four patients having more than one abnormal pattern. A comparison between clinical presentation and imaging category revealed an increased likelihood of having ascites with hepatoperitoneal (*p* < .0001), chylothorax/PB with hepatopulmonary (*p* = .01), and PLE with hepatoduodenal (*p* < .001) connections. Seventy-six patients had a lymphatic intervention, 24% with normal, and 76% with abnormal liver lymphatics. There was no difference in length of hospital stay or mortality between the two groups, but there was a prolonged time to symptom resolution (*p* = .006) and persistent symptoms after 6 months (5% vs 44%, *p* = .002) in the group with abnormal liver lymphatics.

**Conclusion:**

We identified five liver lymphatic imaging categories with a substantial correlation to presenting lymphatic disease. Abnormal imaging patterns correlated with increased morbidity. Evaluation of liver lymphatics should be considered in patients with a systemic lymphatic disease if central lymphatic imaging is normal.

**Key Points:**

• *We identified five liver lymphatic imaging patterns: normal, hepatoperitoneal, hepatomesenteric, hepatopulmonary, and hepatoduodenal.*

• *Imaging patterns were correlated with disease presentation (normal – chylothorax/PB, hepatoperitoneal – ascites/chylothorax, hepatopulmonary – chylothorax/PB, hepatoduodenal – PLE).*

• *Abnormal imaging patterns correlated with increased morbidity.*

**Supplementary Information:**

The online version contains supplementary material available at 10.1007/s00330-021-08098-z.

## Introduction

The main function of the lymphatic system is to remove proteins and fluid from the interstitial space and deliver them back to the venous circulation [1]. The liver is one of the largest contributors of lymphatic fluid, comprising up to 50% of thoracic duct flow [2] and in diseases causing liver congestion, such as heart failure, liver lymphatic flow increases exponentially [3, 4]. There are three categories of liver lymphatic vessels characterized by location: capsular, sublobular, and portal. Liver lymph is generated from hepatic sinusoids in the space of Disse and is connected with the peri-portal space of Mall where the lymphatic capillary vessels begin [2]. The deep portal lymphatics converge at the liver hilum and drain into a complex network of peripancreatic and paraaortic lymph nodes that ultimately connect with the cisterna chyli and thoracic duct [5–9]. The sublobular lymphatics travel along the hepatic veins toward the wall of the inferior vena cava and the capsular lymphatics drain into the mediastinum (convex surface) or toward the liver hilum (concave surface) [5–9]. All of these networks are essential for efficient lymphatic drainage but can become deranged in various pathological conditions.

Patients with congenital heart disease (CHD) or primary lymphatic disorders can develop lymphatic complications, such as protein losing enteropathy (PLE), chylothorax, chylous ascites, and plastic bronchitis (PB), which can lead to significant morbidity and mortality [10–17]. Recent advancements in lymphatic imaging using dynamic MRI have greatly advanced our understanding of the abnormalities present in these patient populations [12, 18]. However, there remains a paucity of literature reports describing the liver lymphatic circulation in humans. Conventional liver lymphangiography has demonstrated abnormalities in patients with liver cirrhosis and chylous ascites [19] as well as PLE and hepatic lymphorrhea [11, 19, 20] and the original liver lymphatic descriptions relied on standard lymphangiography as well as post-mortem analysis [6, 21, 22]. More recently, non-contrast MR liver lymphography has been described as a new technique to noninvasively visualize similar components of the liver lymphatics and changes that occur in portal hypertension [23]. However, this technique only provides a static visualization and does not provide dynamic information that is crucial for possible interventions in systemic lymphatic diseases. Recently, Biko et al. have described the development of intrahepatic dynamic contrast MR lymphangiography (IH-DCMRL) [20]. This technique has several important advantages over conventional liver imaging in that it is 3-dimensional, has good tissue contrast, provides perfusion information, and can visualize well the distal perfused structures.

The purpose of this article is to investigate the anatomy and flow of the efferent liver lymphatic circulation in patients with systemic lymphatic disease.

## Materials and methods

The institutional review board at our institution approved the study with a waiver for informed consent.

### Population

We conducted a retrospective review of imaging and medical records from all patients with lymphatic disease including chylothorax, plastic bronchitis, protein losing enteropathy, chylous ascites, and non-chylous ascites suspected to be of lymphatic origin, who had liver lymphatic imaging with or without lymphatic intervention from May 2014 until April 2019 at our institution. The study included a total of 105 patients. IH-DCMRL was performed as described by Biko et al. [20] using a gadolinium contrast agent in 62 (59%) of the patients. All patients undergoing IH-DCMRL also had intranodal DCMRL (IN-DCMRL). Data collection included patient demographics, clinical presentation, biochemical assessments, lymphatic imaging results, procedure techniques, and outcomes.

### Lymphatic imaging

Liver lymphangiography was performed as described by Clain et al. [21] with the following modifications: Access to the liver lymphatics was achieved using a 25-gauge spinal needle advanced under ultrasound guidance until it apposed a branch of a portal vein. Subsequently, water-soluble iodinated contrast was injected and under fluoroscopy guidance, fine adjustments of needle position were performed until the liver lymphatics were visualized. Conventional fluoroscopic liver lymphangiography was performed with slow hand injection of iodinated water-soluble contrast agent or lipiodol. IH-DCMRL was performed as described by Biko et al. [20] using a gadolinium contrast agent in 62 (59%) of the patients. All patients undergoing IH-DCMRL also had IN-DCMRL. The imaging was subsequently analyzed by two independent reviewers (C.L.S. (interventional cardiologist and lymphatic interventionist, 7y experience) and D.M.B. (radiologist 9y experience)) based on the connections or abnormal flow patterns of the hepatic lymphatics and classified into five different categories: 1) normal (liver connecting to the chylous cistern) as described [5–9], 2) hepatoperitoneal, 3) hepatopulmonary, 4) hepatomesentery, 5) hepatoduodenal. There were four patients who represented multiple abnormal hepatic imaging patterns. Indications for lymphatic imaging included PLE, PB, chylothorax, and ascites.

### Lymphatic interventions and outcomes

Of the patients with lymphatic interventions, their procedures included: Percutaneous embolization with ethiodized oil (lipiodol) or n-butyl cyanoacrylate (n-BCA) injection into abnormal lymphatic vessels, thoracic duct embolization (TDE) or surgical interventions with the creation of lymphovenous anastomoses, pleurodesis, or surgical TDE as previously described [10, 18, 19, 24–27]. Comparisons on the length of stay, number of interventions, time to symptom resolution, failure of symptom resolution after 6 months post-intervention, and death were analyzed based on their hepatic lymphatic anatomy (normal vs. abnormal). Symptom resolution is defined based on their lymphatic disease category as 1) resolution of fluid accumulation (ascites and chylothorax) and removal of drains (if present), 2) absence of airway cast production (in those with PB), and 3) improvement in albumin levels to the normal range (> 3.5 g/dl) (PLE). If multiple underlying lymphatic diseases were present, only those with the resolution of all symptoms were included in this group. If the symptoms persisted beyond six months from their intervention, they were considered a failure to resolve.

### Statistical analysis

Comparisons of baseline preintervention albumin levels between lymphatic disease presentations were done using the Kruskal-Wallis test with Dunn’s multiple comparisons test in Prism v8.1.2 (Graphpad). From the imaging data, differences between imaging findings and lymphatic disease were analyzed using a Pearson chi-squared test (4 × 2 tables) (or 5 × 2 tables for supplemental graph) in Prism v8.1.2 (Graphpad). Differences in clinical outcomes based on lymphatic imaging category (normal vs. abnormal) were statistically analyzed using STATA v14: for the length of stay and time to symptom resolution the Wilcoxon rank-sum test was used and for failure to resolve and death the Fisher’s exact test was performed. The number of interventions was analyzed using the Mann-Whitney test in Prism v8.1.2 (Graphpad).

## Results

### Patient population

The study included 105 patients with demographics summarized in Table 1. Primary clinical presentation consisted of ascites (*n* = 19, 18%), chylothorax (*n* = 27, 26%), plastic bronchitis (*n* = 17, 16%), and PLE (*n* = 42, 40%). Twenty patients (19%) had at least two separate lymphatic diagnoses such as PLE and PB occurring together. Each group had a significant portion of patients with CHD: 16% (3/19) with ascites, 33% (9/27) with chylothorax, 88% (15/17) with PB, and 71% (30/42) with PLE. Several patients presented with pleural or peritoneal drains: ascites (15/19, 79%), chylothorax (22/17, 81%), and PLE (3/42, 7%, peritoneal only).

**Table 1:** Patient Demographics

	Ascites (n = 19)	Chylothorax (n = 27)	PB (n = 17)	PLE (n = 42)
Age at imaging in years (median, IQR, range)	1.2 (IQR 0.4–5.4) (0.2–39)	2.8 (IQR 0.4–8.3) (0.06–17)	9.7 (IQR 5.5–12.9) (1–22)	13.7 (IQR 10.3–18.8) (0.5–29)
Congenital heart diease, # of patients (%)	3 (30%)	9 (33%)	15 (88%)	30 (71%)
Single Ventricle (n)	2	6	14	24
Biventricular repair (n)	1	3	1	6
Albumin g/dL (median, IQR, range)*	3.4 (IQR 2.6–3.7) (2.4–4.8)	3.1 (IQR 2.8–3.8) (2.2–5.1)	4.3 (IQR 3.6–4.8) (2.8–5.1)	2.5 (IQR 1.9–3.4) (1.4–4.3)
Previous lymphatic interventions prior to liver imaging # of patients (%)	7 (37%)	1 (4%)	3 (18%)	5 (12%)
Chest tube(s), Peritoneal drains, or cast production within prior 3 months (PB) (# patients, %)	15 (79%)	22 (81%)	17 (100%)	3 (7%)
Additional lymphatic diagnosis (n)				
Ascites	–	0	1	1
Chylothorax	4	–	1	6
PB	0	0	–	3
PLE	0	2	2	–
Known genetic syndrome/mutation	1 – ARAS 2 – 22q11.2	1 – ARAF 1 – NF1 1 – NF2 1 – Noonan’s 1 – TP53	1 – CHARGE 1 – Noonan	1 – Trisomy 21 1 – Noonan’s 1 – KRAS 1 – Jeune’s 1 – NF1 1 – 12p dup

Legend: PB – plastic bronchitis, PLE – protein losing enteropathy, NF – Neurofibromatosis, IQR – interquartile range (25% - 75%), *Differences are significant between ascites vs PB (*p* < .04), chylothorax vs PB and PLE (*p* = .02 and *p* = .04), and PB vs PLE (*p* < .0001)

### Imaging results

IH-DCMRL (62/105, 59%) and/or IH conventional contrast fluoroscopic lymphangiography (43/105, 41%) from each patient were reviewed and five categories of hepatic lymphatic connections and/or flow patterns were identified: normal (25%, 26/105), hepatoperitoneal (12%, 13/105), hepatopulmonary (10.5%, 11/105), hepatomesenteric (7.5%, 8/105), and hepatoduodenal (41%, 43/105) with four patients (4%) having more than one abnormal hepatic imaging type.

#### Normal liver lymphatics

**Fig. 1:**
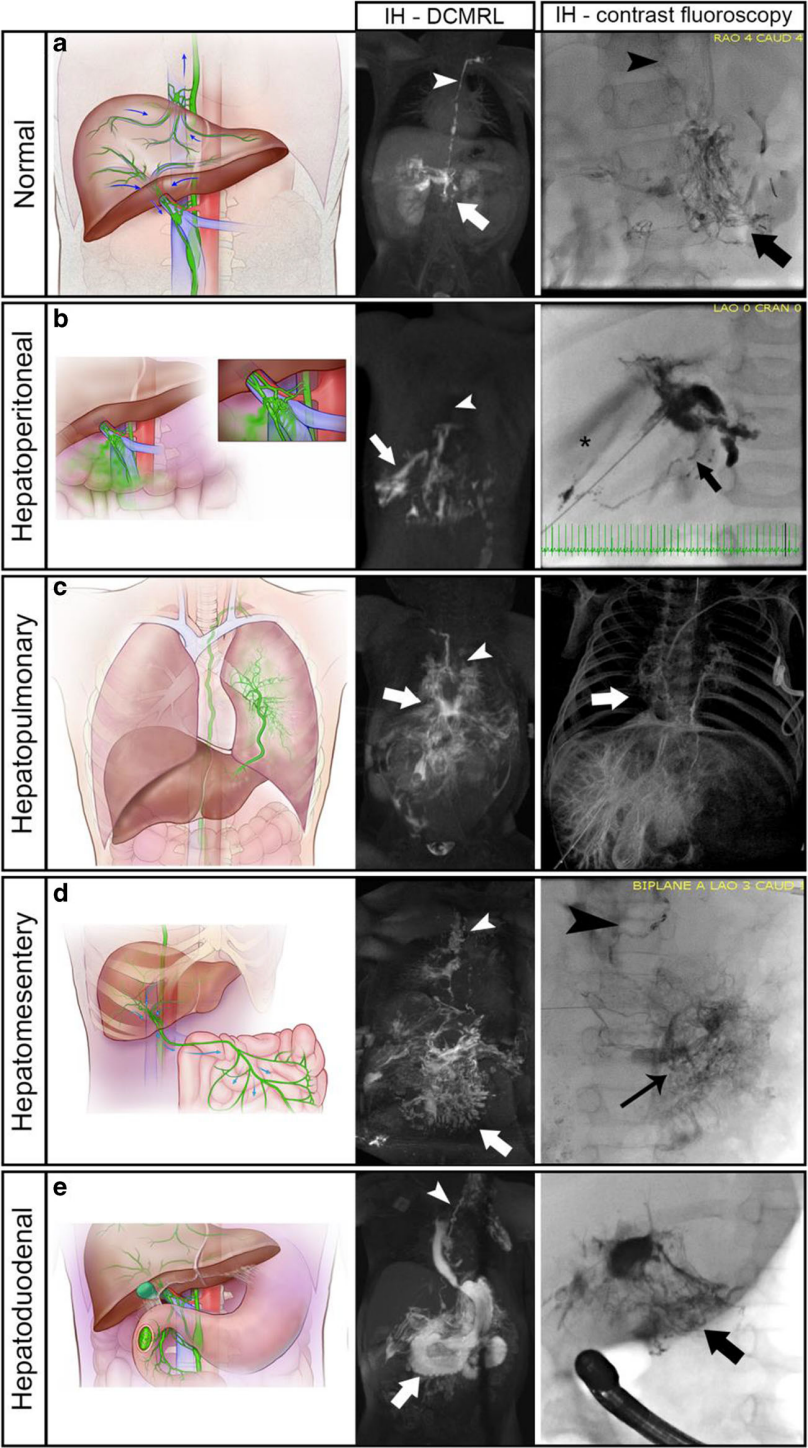
Normal and abnormal hepatic lymphatics with representative maximum intensity projections (MIP) of IH DCMRL and IH contrast fluoroscopy images. Arrowhead represents the normal thoracic duct and arrows denote the abnormal lymphatic connections. (**a**) Normal lymphatic drainage diagram of superficial and deep liver lymphatic drainage. Superficial (capsular) lymphatics directly enter the central TD near the diaphragm while deep (peri-portal) lymphatics course toward the liver hilum and toward the celiac and pancreatic lymphatic networks (arrow) with further connections to the cisterna chylii and thoracic duct (arrowhead). (**b**) Hepatoperitoneal connections with disruption of liver lymphatics after exiting the liver hilum. (**c**). Hepatopulmonary connections from the pericapsular lymphatics of the left liver lobe to the left mainstem bronchus. Arrows represent the abnormal connections with the ductal remnant noted with an arrowhead (**d**) Hepatomesenteric connections from the liver to the mesentery with intact TD and pulmonary lymphatic perfusion (**e**) Hepatoduodenal representation of the liver lymphatics as they exit the liver hilum and course to the inner curvature of the 1st – 3rd portions of the duodenum (arrows) with significant reflux into the stomach and esophagus (asterisk) with propagation forward to the proximal jejunum

**Fig. 2:**
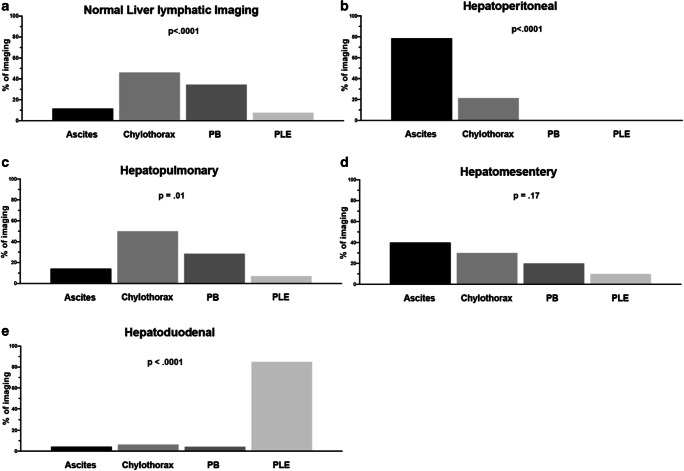
Hepatic lymphatic imaging compared to lymphatic disease presentation. Percentage of imaging with (**a**) normal, (**b**) hepatoperitoneal, (**c**) hepatopulmonary, (**d**) hepatomesentery, and (**e**) hepatoduodenal as compared to presentation with ascites, chylothorax, PB, and PLE. Note the significance of the imaging compared to the likelihood of having the underlying lymphatic disease type

Twenty-six of the 105 patients (25%) including 12% (3/26) with ascites, 46% (12/26) with chylothorax, 35% with PB (9/26), and 7% (2/26) with PLE had drainage patterns consistent with normal lymphatic connections to the thoracic duct (Fig. 1a). This distribution was statistically significant (*p* < .0001) with a higher likelihood of having chylothorax or PB (Fig. 2a, Fig. S1). Since the normal connections allowed the opacification of the central thoracic duct, we observed abnormal pulmonary lymphatic perfusion originating from the TD in all patients with PB and chylothorax. The two patients with clinical PLE had normal intrahepatic and intranodal lymphangiograms and did not have congenital heart disease.

#### Hepatoperitoneal

Fourteen of the 105 patients (12%) including 78% (11/14) with ascites, 21% (3/14) with chylothorax, and one patient with both had a direct connection of the liver lymphatics to the peritoneal compartment with or without normal connections to the TD (Fig. 1b). One patient in this group had multiple abnormal hepatic connections (hepatopulmonary, hepatomesentery, and hepatoduodenal). There were no patients with PB or PLE in this category. This distribution was statistically significant (*p* < .0001) with a higher likelihood of having ascites or chylothorax (Fig. 2b, Fig. S1). Three of the patients with ascites had recent Nissen fundoplication with gastrostomy tube placement and IH-DCMRL demonstrated a peritoneal leak in 2/3 patients and all three with normal connections to the central lymphatic system.

#### Hepatopulmonary

Fourteen of the 105 patients (13%) including 14% (2/14) with ascites, 50% (7/14) with chylothorax, 29% (4/14) with PB, and 7% (1/14) with PLE, showed direct drainage from the liver lymphatics via abnormal connections to the perihilar or intrapulmonary lymphatics from the deep hepatic lymphatics through continuity with the subcapsular lymphatics (Fig. 1c). Three patients with hepatopulmonary connections also demonstrated other abnormal connections: two with hepatoduodenal, and one with hepatopulmonary, hepatomesentery, and hepatoduodenal. Combined, 85% (11/13) of patients had retrograde pulmonary lymphatic perfusion (PLP) (known etiology of chylothorax and PB). This distribution was statistically significant (*p* = .01) with a higher likelihood of having chylothorax and/or PB (Fig. 2c, Fig. S1).

#### Hepatomesenteric

Ten of the 105 patients (9.5%) including 40% (4/10) with ascites, 30% (3/10) with chylothorax, 20% (2/10) with PB, and 10% (1/10) with PLE showed retrograde perfusion of the mesenteric lymphatics from the liver in addition to normal connections to the central lymphatic system (Fig. 1d). Two patients with hepatomesenteric connections also demonstrated other abnormal connections: one with hepatoduodenal, and one with hepatoperitoneal, hepatopulmonary, and hepatoduodenal. This distribution was not statistically significant across all disease categories (*p* = .17) (Fig. 2d, Fig. S1).

#### Hepatoduodenal

The largest group had 47 of the 105 patients (45%) including 4.5% (2/47) with ascites, 6% (3/47) with chylothorax, 4.5% (2/47) with PB, and 85% (40/47) with PLE, had abnormal liver to duodenal lymphatic connections (or flow) (Fig. 1e). A significant subpopulation of patients with PLE had single ventricle congenital heart disease (24/42, 57%). Four patients with hepatoduodenal connections also demonstrated other abnormal connections: two with hepatopulmonary, one with hepatomesenteric, and one with hepatoperitoneal, hepatopulmonary, and hepatomesentery. This distribution was statistically significant (*p* < .0001) with a higher likelihood of having PLE (Fig. 2e, Fig. S1).

### Lymphatic intervention

Seventy-six patients (72%) underwent lymphatic intervention including 18 patients (24%) with normal hepatic lymphatics, but with other observed abnormalities in the lymphatics system visualized by IH DCMRL and/or IN DCMRL, and 58 (76%) with abnormal hepatic lymphatics (Table 2). The normal group included chylothorax or PB (17/18) and one patient with chylous ascites. Ten of the 18 patients (56%) had percutaneous interventions with TD embolization (TDE) and 8 (44%) had selective embolization of abnormal lymphatic networks. The 58 patients in the abnormal liver lymphatic group consisted of each disease presentation with 9 (16%) ascites, 9 (16%) chylothorax, 8 (14%) PB, and 32 (55%) with PLE. The patients had the following imaging classification: 10 (17%) hepatoperitoneal, 6 (10%) hepatopulmonary, 6 (10%) hepatomesenteric, 33 (57%) hepatoduodenal, 1 (2%) with all types of abnormal imaging (hepatoperitoneal, hepatopulmonary, hepatomesentery, and hepatoduodenal), 1 (2%) with hepatopulmonary and hepatoduodenal, and 1 (2%) with hepatomesentery and hepatoduodenal. Interventions within each group included: percutaneous embolization of abnormal lymphatic channels (*n* = 51), surgical ligation (*n* = 6), and surgical lymphovenous anastomosis (*n* = 1).

**Table 2:** Abnormal hepatic lymphatic drainage predicts worse outcome in patients undergoing lymphatic interventions

	Normal (*n* = 18)	Abnormal (*n* = 58)	*p* value
# of interventions per patient (Median, IQR, range)	1.3 IQR (1–1) (1–4)	2.0 IQR (1–3) (1–6)	.001
Patients without symptom resolution at 6 months post procedure (n, %)	1(5.5%)	28 (44.4%)	.002
death (n, %)	3 (17%)	11 (19%)	> .99

**Fig. 3:**
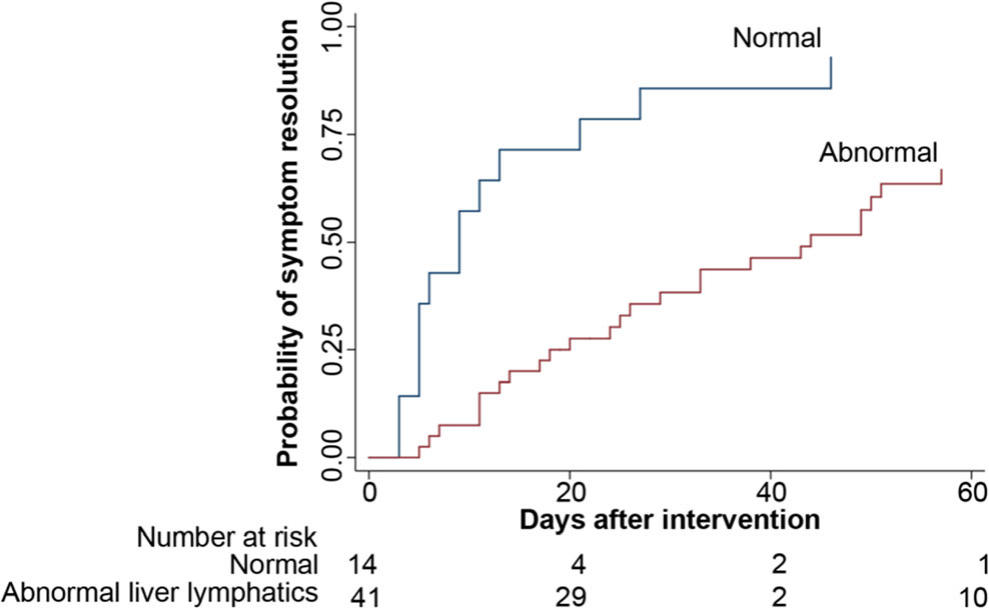
Kaplan Meir curve demonstrating the time to symptom resolution between normal and abnormal liver lymphatics. The median time for resolution is 9 days in the normal group and 44 days in the abnormal group (*p* = 0.0001)

There was no difference in the median time from intervention to hospital discharge between the two groups (Kaplan Meier analysis, *p* = .43), with the time to 50% of patients being discharged of 11 days (range 2–215) in the normal group vs 20 days (range 2–282) in the abnormal group. However, the median number of interventions was higher in patients with abnormal liver lymphatics (1.0 vs 2.0, *p* = .001) (Table 2). The presence of abnormal liver lymphatics was also associated with a longer time to symptom resolution and a higher probability of non-resolution of symptoms. The time to 50% of patients that were symptom-free was 9 days for normal and 33 days for abnormal liver lymphatics (Wilcoxon rank-sum, *p* = .002) (Fig. 3) but there was a significantly higher percentage of patients that did not have a resolution of their symptoms up to 6 months post-procedure (5.5% vs 44.4%, *p* = .002). There was not a statistically significant difference in mortality (17% vs 19%, *p* > .99) (Table 2). None of the reported deaths were procedure-related and included respiratory failure (*n* = 6), sepsis (*n* = 3), heart failure (n = 3), and multisystem organ failure (*n* = 2).

## Discussion

Using novel lymphatic imaging techniques, this study has identified an under-appreciated role of liver lymphatics in systemic lymphatic diseases. Five different imaging categories (normal, hepatoperitoneal, hepatopulmonary, hepatomesenteric, and hepatoduodenal) were identified and further observed that they were substantially correlated with disease presentation (normal – chylothorax/PB, hepatoperitoneal – ascites/chylothorax, hepatopulmonary – chylothorax/PB, hepatoduodenal – PLE). In addition, the presence of abnormal patterns correlated with increased morbidity of their disease.

Liver lymphatics are a major contributor to central lymphatic flow and while their role in systemic diseases such as liver cirrhosis and heart failure have been studied previously, there is little information about the involvement in systemic lymphatic disorders. This has in part been limited by the inability to clinically visualize the liver lymphatics. However, with recent advancements in imaging modalities and techniques, we can now visualize organ-specific lymphatic connections such as those from the liver [20]. We have used these new modalities and now report on the role of the liver lymphatics in chylothorax, ascites, PB, and PLE.

The identification of normal hepatic lymphatic connections has been predominately studied from traditional lymphangiography and anatomic studies and have been limited in their ability to describe a 3D relationship and distal connections with the central lymphatic system. In our cohort, we had a significant number of patients with anatomically normal connections via perihepatic/peripancreatic/paraaortic lymphatic vessels and lymph nodes that drain into the central thoracic duct. This is the largest known cohort to show the detail of these connections. Furthermore, using the normal anatomy as a baseline we identified four types of abnormal connections.

We identified that abnormal hepatoperitoneal connections were most commonly observed in patients with underlying ascites consistent with previously reported case studies [11, 19, 28]. In this group, standard intranodal lymphangiography did not elucidate the mechanism and was only visualized with hepatic lymphatic imaging. While there have been previous reports of abnormal hepatic lymphatic drainage in ascites, our results indicate this could be a much more common mechanism than previously thought and would suggest considering an evaluation of liver lymphatics in all patients with ascites.

Previously, a few different etiologies of chylothorax and PB have been described and predominately originate from retrograde PLP from the central lymphatics [10, 12, 18, 25, 27]. However, there have been several patients in our study that have PB and/or chylothorax with no significant PLP identified from the thoracic duct, suggesting another possible lymphatic source. With organ-specific intrahepatic lymphangiography, we identified a subset of patients who have abnormal connections from the liver lymphatics with direct connections to the pulmonary lymphatics bypassing the central lymphatic system. Liver lymphatic imaging should likely be performed in all patients with chylothorax and PB when central lymphatic imaging is normal.

One unique group that has not been previously reported was the abnormal connection/flow from the hepatic lymphatics to the mesentery (hepatomesentery). It is unclear how these abnormal connections or reversal of flow develops or how this affects the drainage of intestinal lymphatics. It could result in bowel edema or even potentially ascites with the dilated and congested lymphatics leaking into the peritoneum. However, in our small group of patients with these findings, there was no clear association with any specific type of lymphatic disorder.

Subsets of PLE, particularly patients that have congenital heart disease, are known to have abnormal hepatoduodenal connections that were seen in a small series of patients [11, 19, 28]. In our larger cohort of 47 patients with PLE, hepatoduodenal connections were almost pathognomonic in our study population. This will need further investigation, but it does suggest that abnormal hepatic connections could be much more common in this patient population than previously thought.

In addition to the identification of the four abnormal compartment connections from the liver lymphatics in disease states, we have also determined that the presence of these connections has a clinically significant implication in their disease course. In patients who underwent lymphatic interventions, those with abnormal liver lymphatics required more interventions, had a longer time for symptom resolution, and were more likely to have a failure of treatment with symptoms present 6 months after the intervention. This could be caused by the delayed diagnosis of liver involvement in disease as there were a significant number of patients that had previous lymphatic interventions in this study. In addition, there may be individual differences between each subgroup of abnormal lymphatics and disease presentation that this study was not powered to detect. Even with the early identification and intervention, it is still likely there are differences between this etiology of these diseased vessels, such as the number/density/location of the abnormal lymph vessels as well as their response post-treatment that are unknown.

## Limitations

This is a single-center retrospective review of a limited number of patients referred for evaluation of lymphatic diseases that also included evaluation of liver lymphatics. This represents a selection bias and may not be representative or generalizable to all types of lymphatic disease. However, this does not affect the identification of the observed hepatic lymphatic drainage patterns described here, but may not truly reflect the correlation between imaging findings and disease presentation in a different population. In addition, not all patients had IH DCMRL and were diagnosed with IH contrast lymphangiography, either due to MRI contraindications or prior to our IH DCMRL experience. IH contrast lymphangiography is less sensitive to subtle abnormalities and may not identify all patients with abnormal perfusion, but does not limit the identification of the same imaging categories.

## Conclusion

We identified five different imaging categories that demonstrated a substantial correlation to presenting systemic lymphatic disease. The presence of abnormal imaging patterns correlated with increased morbidity of their disease and warrants further investigation. Liver lymphatics should be evaluated in all patients with a systemic lymphatic disease if central lymphatic imaging is normal.

## Supplementary Information

ESM 1 (DOCX 179 kb)

## Abbreviations

CHD: Congenital heart disease
DCMRL: Dynamic contrast magnetic resonance lymphangiography
IH: Intrahepatic
MIP: Maximum intensity projection
NF: Neurofibromatosis
PB: Plastic bronchitis
PLE: Protein losing enteropathy
PLP: Pulmonary lymphatic perfusion
TD: Thoracic duct
TDE: Thoracic duct embolization

## Declarations

### Guarantor

The scientific guarantor of this publication is Yoav Dori MD, PhD.

### Statistics and biometry

One of the authors has significant statistical expertise.

### Study subjects or cohorts overlap

Some study subjects or cohorts have been previously reported.

We have included patients that were published in other technique papers or non-imaging based papers. These were our original small case series/technique papers describing intrahepatic MRL and intramesenteric MRL, and a small case series from our original description of PLE (reference [11], 20, and European Radiology 2020 Oct;30(10):5777–5784). Together these papers had 29 patients, which is a much smaller number of patients than this study that includes 105 patients. In addition, this study is a more comprehensive description of liver lymphangiography by expanding on these techniques to describe this larger and broader patient population and correlates both pathologic patterns with disease presentation as well as disease outcome after intervention that has not been previously described.

**Table:**

**# of patients**	**Publication**	**Journal**	**Pubmed ID**
8	Protein-Losing Enteropathy in Patients With Congenital Heart Disease	J Am Coll Cardiol. 2017 Jun 20;69(24):2929–2937	28,619,193
6	Intrahepatic dynamic contrast MR lymphangiography: initial experience with a new technique for the assessment of liver lymphatics	European Radiology 2019. Oct;29(10):5190–5196	30,887,210
15	Intramesenteric dynamic contrast pediatric MR lymphangiography: initial experience and comparison with intranodal and intrahepatic MR lymphangiography	European Radiology 2020 Oct;30(10):5777–5784	32,462,442

### Methodology

• retrospective

• observational

• performed at one institution
