# Supplementary material for: Liver lymphatic anatomy and role in systemic lymphatic disease
Source: Eur Radiol. 2021 Jun 24;32(1):112–21. doi: 10.1007/s00330-021-08098-z (PMC8660706; doi:10.1007/s00330-021-08098-z)
Supplement: Supplementary file 1 — (DOCX 179 kb) [file 330_2021_8098_MOESM1_ESM.docx]

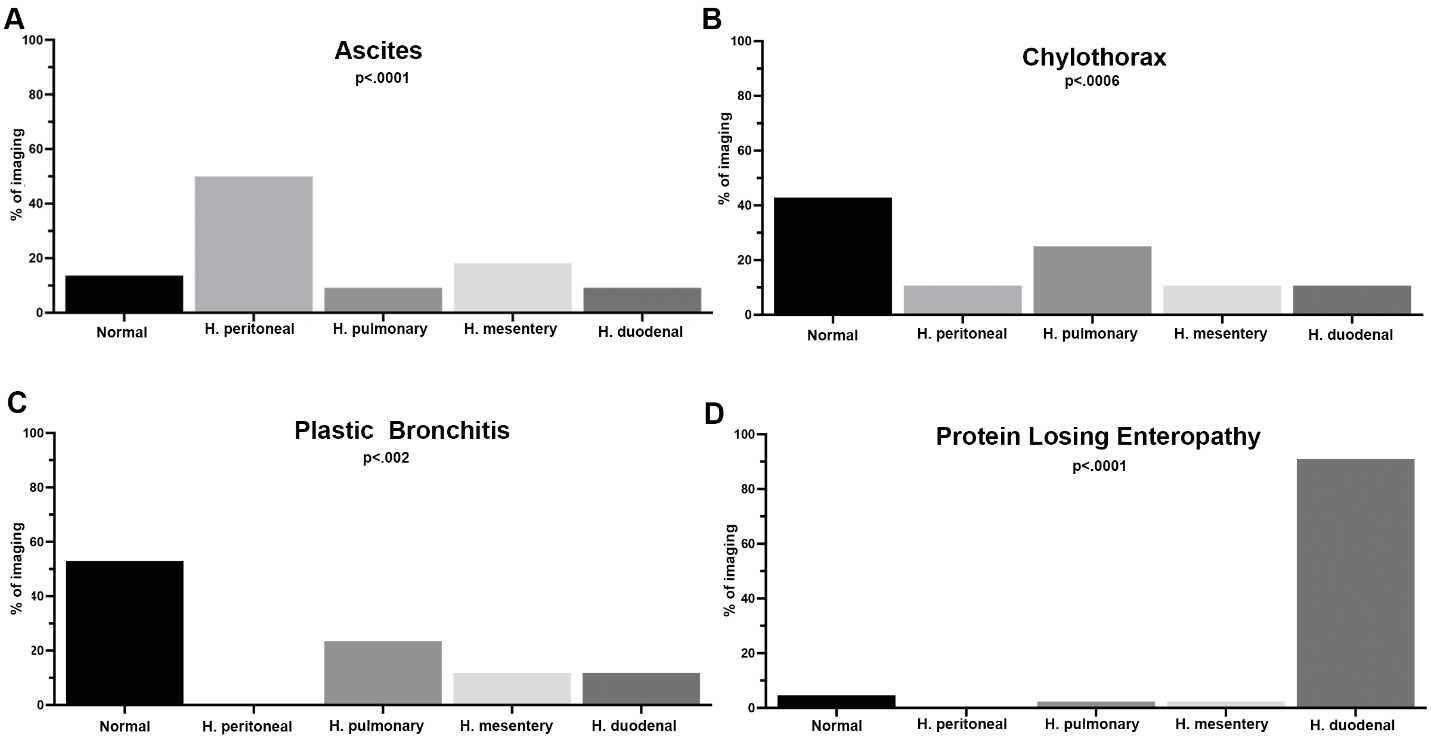


Figure S1: Lymphatic disease presentation compared to hepatic lymphatic imaging. Percentage of imaging with (A) ascites, (B) chylothorax, (C) plastic bronchitis, and (D) protein losing enteropathy as compared to lymphatic imaging finding, hepatoperitoneal, hepatopulmonary, hepatomesentery, and hepatoduodenal. Note the association of imaging type with disease presentation.
